# Therapeutic effects and mechanisms of berberine on enteritis caused by *Salmonella* in poultry

**DOI:** 10.3389/fmicb.2024.1458579

**Published:** 2024-11-27

**Authors:** Li Yang, Jingwen Sun, Tong Yang, Xumei Zhang, Chenghui Xu, Yaya Wei, Yongshuai Li, Yan Zhao, Sheng Zhang, Quanxin Wu, Huijun Shi, Qiang Fu, Lining Xia

**Affiliations:** Xinjiang Key Laboratory of Herbivore Drug Research and Creation, College of Veterinary Medicine, Xinjiang Agricultural University, Urumqi, Xinjiang, China

**Keywords:** berberine, salmonellosis, intestinal flora, transcriptome, broiler

## Abstract

The present study aimed to investigate the therapeutic effects of berberine (BBR) on *Salmonella* enteritis in broiler chickens and to elucidate its mechanisms of action preliminarily. Blood samples were collected from 21- to 35-day-old Sanhuang male chicks to measure immune and biochemical indicators and to calculate the organ coefficients for the liver, spleen, bursa of Fabricius, and thymus. The caecal microbiota was analysed through 16S ribosomal RNA (rRNA) gene sequencing, and transcriptome sequencing was conducted. Compared with the positive control group (S), the berberine-treated group (BS) presented increased serum immunoglobulin M (IgM) levels, serum IgG levels, and total antioxidant capacity; berberine ameliorated the increase in the thymus index caused by *Salmonella* administration. The addition of berberine to the diet increased the abundance of beneficial bacterial genera, including *Bacteroides* and *Lactobacillus*. It also decreased the abundance of harmful bacterial genera, including *Faecalibacterium* and *Streptococcus*. Transcriptome analysis revealed that gene expression in the S and BS groups was associated with T cell selection and B cell receptor signalling pathways, which are enriched primarily in multiple immune-related signalling pathways, including the B cell receptor signalling pathway, NF-*κ* B signalling pathway, intestinal immune network for IgA production, asthma, and African trypanosomiasis. The significantly expressed genes included ATAD5, ERP29, MGST2, PIK3CA, and HSP90AA1. The present study demonstrated that berberine has a good therapeutic effect on *Salmonella* infection in chicks, as it inhibits the occurrence and development of *Salmonella*-induced intestinal inflammation by regulating the balance of the gut microbiota and the expression of related genes, including ATAD5, ERP29, MGST2, PIK3CA, and HSP90AA1.

## 1 Introduction

*Salmonella*—a widely distributed Gram-negative enteric bacterium—has long been a focal point of concern in global public health. Since its first description by the American physician, Daniel Elmer Salmon, in 1885, multiple serotypes within the genus *Salmonella* have been identified. However, more than 2,500 different serotypes have been identified (Zhang Wen-Cheng et al., 2019) as time progressed. As an important zoonotic pathogen, *Salmonella* can cause various clinical syndromes in both animals and humans, including enteric fever (typhoid fever), gastroenteritis or diarrhoea, bacteraemia, and chronic asymptomatic carriage, which are collectively referred to as salmonellosis (Bao et al., 2022; Johari et al., 2019). *Salmonella* infection—typically transmitted through ingesting contaminated food or water—poses a severe threat to human health, particularly for children, elderly individuals, and individuals with compromised immune systems. Studies have indicated that *Salmonella* causes approximately 90 million cases of gastroenteritis globally each year, resulting in about 155,000 deaths. It is estimated that about 10% of these cases are due to direct contact with animals (Galán-Relaño et al., 2023). Globally, *Salmonella* is one of the leading causes of foodborne illness (Robinson, 1970). According to data from the Centres for Disease Control and Prevention (CDC), there are approximately 1.35 million cases of human *Salmonella* infections in the United States each year. Additionally, the European Food Safety Authority (EFSA) and the European Centre for Disease Prevention and Control (ECDC) reported that in 2018, *Salmonella* was responsible for nearly one-third of all foodborne diseases in Europe (Junwei, 2021). *Salmonella* not only poses a threat to human health but also has a significant effect on the livestock and poultry industry. *Salmonella* infections can lead to substantial mortality in chicks, a decrease in egg production, a reduction in the hatchability rate of breeder eggs, and an increase in the rate of weak chicks (Liang et al., 2019). Additionally, *Salmonella* can contaminate eggs through vertical transmission, affecting hatchability rates and the health of chicks, leading to significant economic losses in the poultry industry (Zhai Wendong, 2011).

Currently, antibiotic therapy remains the primary means of controlling *Salmonella* infections. However, with the extensive use and misuse of antibiotics, the resistance of *Salmonella* to multiple antibiotics is continuously increasing. Studies have shown that up to 36 mutational genes have emerged in *Salmonella*, conferring varying degrees of resistance to 14 types of antibiotics, including cephalosporins, penicillin, and tetracycline (Zhao et al., 2023). In a study conducted in the northern region of Jiangsu, *Salmonella* strains isolated from parent stock and commercial broilers of white feather meat chickens exhibited resistance to multiple antibiotics (Fu et al., 2020). Traditional antibiotic treatments are inadequate in the face of the increasing problem of antibiotic resistance. Therefore, it is crucial to identify new therapeutic strategies and medications.

Berberine (BBR) is a quaternary protoberberine alkaloid isolated from traditional Chinese medicinal plants, such as Coptis chinensis (Huang Lian). Berberine is widely distributed in the plant kingdom and has been identified in approximately four families and 10 genera (Dan, 2022; Fang, 2020). Berberine exhibits potent pharmacological activities, and its medicinal applications have expanded significantly. Initially used clinically for the treatment of diarrhoea, the scope of the medical use of berberine now extends to include antimicrobial (Zhang and Shen, 2023a; Zhang and Shen, 2023b), anti-inflammatory, antitumour (Cheng et al., 2022; Song et al., 2019), blood sugar and lipid modulation (Yuting Chen et al., 2022), diabetes treatment (Shan et al., 2013; Xiao et al., 2021), and adjuvant therapy for liver diseases (Li et al., 2017). Due to its unique chemical structure and diverse biological activities, BBR can act on multiple signalling pathways and target various therapeutic sites (Feng et al., 2012; Izadparast et al., 2022). Additionally, many studies have indicated that berberine has significant efficacy in treating intestinal damage induced by dextran sulphate sodium (DSS) (Dong et al., 2022) and trinitrobenzene sulphonic acid (TNBS) (Li et al., 2015), as well as in genetically modified animal models and models of intestinal damage induced by lipopolysaccharide (LPS) (Guo et al., 2018).

A study mentioned that the combination of berberine with antibiotics can reduce the formation of biofilms in multidrug-resistant bacterial strains and decrease the multidrug resistance of *Salmonella* by inhibiting messenger RNA (mRNA) expression (Shi et al., 2018). Furthermore, research by Xiao-die Cui and colleagues found that the combination of berberine and ethylenediaminetetraacetic acid (EDTA) significantly restored the sensitivity of *Salmonella* and *Escherichia coli* resistant to colistin (Cui et al., 2024). Our previous research revealed that berberine alleviates the inflammation caused by *Salmonella* lipopolysaccharide stimulation in IPEC-J2 cells (Yang et al., 2022). These studies provide preliminary evidence of berberine’s potential roles and mechanisms in combating *Salmonella* infections. However, there is currently a lack of research on treating *Salmonella* infections with berberine, and there have been no reports on whether berberine can treat avian salmonellosis caused by *Salmonella* and the potential mechanisms involved. Therefore, the present study utilised poultry infected with *Salmonella* to investigate the mechanisms of action of berberine treatment for *Salmonella* infection by examining growth performance, histopathology, biochemical indicators, and the gut microbiota through 16S ribosomal RNA (rRNA) analysis and transcriptomics. The present study aimed to identify critical targets for treating *Salmonella* infection from transcriptomics results, explore the specific mechanisms of BBR in treating this infection, and provide a new traditional Chinese medicine for the *Salmonella* disease.

## 2 Materials and methods

### 2.1 Preparation of BBR and *Salmonella*

Berberine (catalogue number: 110713) was purchased from the China Food and Drug Administration (CFDA). *Salmonella enteritidis* (*Salmonella enterica* subsp. *enterica serovar*, Typhimurium strain LT2) was provided by Professor Su Zhanqiang from Xinjiang Agricultural University.

### 2.2 Animals and management

All the experimental procedures adhered to the regulations set by the Animal Care and Use Committee of the College of Veterinary Medicine at Xinjiang Agricultural University.

A total of 120 1-day-old healthy Sanhuang male chickens with similar body weights were purchased from Tiankang Biological Company, Wuhan, Hubei Province, China. The chickens were randomly divided into four groups, with six replicates in each group, and each replicate consisted of five Sanhuang male chickens. All the chicks were housed in an animal facility with adequate lighting, free access to food and water, and a controlled temperature environment. The rearing temperature was maintained at 35–40°C for the first 3 days, at 32–33°C from day 4 to day 7, at 28–32°C from day 8 to day 21, and at room temperature (25°C) after day 21. The relative humidity of the rearing environment for the chicks throughout the experiment was maintained at 55%. The rearing experiment was conducted at the College of Animal Medicine, Xinjiang Agricultural University. During their growth period, all chicks were vaccinated with an inactivated infectious bursal disease vaccine or an inactivated Newcastle disease vaccine. The basal diet for the chickens was purchased from Tiankang Biological Company, and the diet’s nutritional content is shown in Table 1. The standards met the requirements set by the National Research Council (NRC) and satisfied the daily nutritional needs of Sanhuang male chickens.

**Table 1:** Ingredients and composition of basal diet.

Item	Starter (1–14 D)	Grower (15–35 D)
Ingredients		
Corn, yellow	54.9	57.5
Soybean meal	37.5	34.2
Soybean oil	3.3	4.0
Dicalcium phosphate	2.45	2.34
Limestone	1.50	1.10
Common salt	0.4	0.3
Vitamin premix^a^	0.25	0.25
Mineral premix^b^	0.25	0.25
DL-Methionine	0.17	0.10
Nutrient composition		
ME (kcal/kg)	3,006	3,105
CP (%)	23.04	21.57
Methionine (%)	0.49	0.36
Lysine (%)	1.18	1.01
Calcium (%)	0.97	0.85
Nonphytate P (%)	0.48	0.44

a Supplied per kilogram of diet: vitamin A (retinyl acetate), 15,000 IU; vitamin D3, 5,000 IU; vitamin E (DL-*α*-tocopheryl acetate), 80 mg; vitamin K, 5 mg; thiamin, 3 mg; riboflavin,10 mg; pyridoxine, 5 mg; vitamin B12, 0.02 mg; niacin, 70 mg; folic acid, 2 mg; biotin, 0.4 mg; pantothenic acid, 20 mg.

b Supplied per kilogram of diet: Cu, 16 mg; I, 1.5 mg, Co, 500 μg; Se, 350 μg; Fe, 60 mg; Zn, 100 mg; Mn, 120 mg; Mo, 1 mg.

The experiment utilised a 2 × 2 factorial design, with the following groups: a control group (C), in which the diet did not contain berberine and no *Salmonella* was administered orally; a positive control group (S), in which the diet did not contain berberine but *Salmonella* was administered orally; a berberine group (B), in which the diet was supplemented with berberine without oral administration of *Salmonella*; and a berberine-treated group (BS), in which the diet was supplemented with berberine and *Salmonella* was administered orally. The feed of groups B and BS was supplemented with 120 mg/kg berberine (Feng et al., 2021), which was freshly provided. *Salmonella* was dissolved in sterile physiological saline, and at 9 days of age, groups S and BS were orally administered 1.2 × 10^9^ colony-forming units (CFU) of *Salmonella* for 3 consecutive days (Lamichhane et al., 2024), whereas groups C and B received an equal volume of physiological saline.

### 2.3 Sample collection and procedures

The chicks were weighed after an overnight fast at 1 day, 14 days, 20 days, and 34 days of age, and the remaining feed amount for each group was recorded. Based on the body weight and feed quantity obtained, the average daily gain (ADG), average daily feed intake (ADFI), and feed conversion ratio (FCR) for the chickens at 15 days, 21 days, and 35 days of age were calculated. At 21 and 35 days of age, five chicks were randomly selected from each group for necropsy. Blood samples (3–5 mL) were collected from the wing vein of the chicks, and the blood was allowed to stand at room temperature for 30 min before being centrifuged at 6,000 rpm for 15 min in a refrigerated centrifuge. The obtained serum was stored at −20°C for future use. After blood collection, the chicks were weighed, euthanized, and subjected to necropsy.

The liver, spleen, and bursa of Fabricius were collected; to retrieve the thymus glands on both sides, the thoracic cavity was opened. After removing the fascia and adipose tissues, the blood was collected and the organs were weighed. The weights were recorded, and the immune organ index—the ratio of the organ weight to the live body weight—was calculated. Additionally, at 21 days of age, the caecum and its contents were collected from the chicks for examination. A portion of the caecum was washed in ice-cold PBS and fixed in 10% formalin solution for 24 h, after which it was sent to the Wuhan Sevilla: Wuhan Sevilla Company, Wuhan, Hubei Province, China for HE staining and tissue sectioning. The remaining caecal tissue was cleaned with RNase-free water, frozen in liquid nitrogen for 30 min, and stored at −80°C. The caecal contents were also frozen in liquid nitrogen for 30 min and stored at −80°C. From each group, three random caecal samples and three caecal content samples were collected and sent to the Shanghai Majorbio Company, Shanghai, China for transcriptome sequencing and 16S rRNA sequencing of the gut microbiota.

### 2.4 Determination of *Salmonella* content in faeces

Faeces were collected from 35-day-old broilers. *Salmonella* DNA was extracted from the faeces via the TaKaRa MiniBEST Universal Genomic DNA Extraction Kit (version 5.0). Absolute quantification was performed via real-time fluorescent quantitative polymerase chain reaction (PCR) (primers invA-F: ACCAGTCGTCTTATCTT GATTGAAG and invA-R: TCATCGCACCGTCAAAGGAA). The copy number of *Salmonella* in each group was calculated based on the absolute quantification standard curve obtained from a known concentration standard (invA plasmid).

### 2.5 Biochemical index analysis

From each group, five serum samples were randomly collected for the detection of immunoglobulins (IgA, IgM, and IgG), antioxidant indicators (alkaline phosphatase activity and total antioxidant capacity), high-density lipoprotein (HDL), low-density lipoprotein (LDL), triglyceride, and total cholesterol contents according to the manufacturer’s instructions. The IgA ELISA Kit (YX-E21412C), IgM ELISA Kit (YX-E21856C), and IgG ELISA Kit (YX-E21430C) were purchased from Shanghai Youxuan Biotechnology Co., Ltd., Shanghai, China and the Total Antioxidant Capacity Assay Kit (A015-2-1), Alkaline Phosphatase Assay Kit (A059-2-2), High-Density Lipoprotein Assay Kit (A112-1-1), Low-Density Lipoprotein Assay Kit (A113-1-1), Triglycerides Assay Kit (A110-1-1), and Total Cholesterol Assay Kit (A111-1-1) were obtained from the Nanjing Jiangcheng Bioengineering Research Institute, Nanjing, China.

### 2.6 Histopathological examination

The caecum of the chicks at 21 days of age was sent to the Wuhan Sevilla Company for tissue sectioning and haematoxylin and eosin (HE) staining. For each sample, three representative intestinal villi and crypts were collected, and the villus height (V) and crypt depth (C) were measured via the ImagePro-Plus 7.0 image analysis software (Media Cybernetics, Rockville, MD, United States) (Unit: μm).

### 2.7 16S rRNA sequencing and bioinformatics analysis

During the necropsy of 21-day-old chicks, the caecal contents were removed under sterile conditions and quickly frozen in liquid nitrogen. After 30 min, the contents were stored at −80°C. Three samples were randomly selected from each group and sent to Shanghai Majorbio Bio-Pharm Technology Co., Ltd., Shanghai, China. for 16S rRNA sequencing of the gut microbiota. Sequencing was performed via the Illumina MiSeq platform (Illumina, Inc), and initial data quality control was conducted via Fastp (version 0.19.6) (HaploX Biotechnology and Shenzhen Institutes of Advanced Technology, Chinese Academy of Sciences), which removes sequences shorter than 50 base pairs. UPARSE (version 7.0.1090) (Robert C. Edgar) was used for operational taxonomic unit (OTU) clustering analysis. The Ribosomal Database Project (RDP) classifier (version 2.11) (Bayesian algorithm) (Michigan State University) was used to conduct a taxonomic analysis of the representative sequences of OTUs at a 97% similarity level in the database and to calculate the community species composition of each sample.

### 2.8 Transcriptome sequencing and bioinformatics analysis

During the necropsy of 21-day-old chicks, the caecum was removed under sterile conditions, washed in RNase-free water, and frozen in liquid nitrogen. After 30 min, the caecum was stored at −80°C. From each group, three samples were randomly selected and sent to Shanghai Majorbio Bio-Pharm Technology Co., Ltd. for sequencing on the Illumina MiSeq platform. Fastp (version 0.19.6) was used to remove low-quality reads, adapters, and sequences shorter than 50 base pairs. The quality of the post-QC data was further assessed and statistically analysed, with Q20 (%) and Q30 (%) used as metrics for quality evaluation and alignment with reference sequences. RNA-Seq by Expectation–Maximisation (RSEM version 1.3.3) (Bo Li Colin Dewey and Peng Liu) was used for expression analysis between samples. DESeq2 (version 1.24.0) (Bioconductor) was used for differential expression analysis of genes and transcripts between samples, with a threshold of |log_2_FC| ≥ 1.000, where FC denotes fold change and a *p* < 0.05. Goatools software (version 0.6.5) (Xiaoqian Jiang and Yu Lin), China was used for gene ontology (GO) enrichment analysis of the gene set to determine the main GO functions of the genes in the set. Fisher’s exact test was applied, and a corrected *p* < 0.05 indicated significant enrichment of the GO function. (R scripts) (R Core Team) were used to perform the Kyoto Encyclopedia of Genes and Genomes (KEGG) pathway enrichment analysis on the gene set, with the same computational principles as those used for the GO functional enrichment analysis. A corrected *p* < 0.05 was considered to indicate significant enrichment of the KEGG pathway function.

### 2.9 Quantitative real-time PCR

Based on the results of the transcriptome sequencing, the key genes whose expression significantly differed between the positive control group (S) and the berberine-treated group (BS) were screened, and the expression levels of five genes related to the treatment of enteritis caused by *Salmonella* were detected via qRT-PCR. RT-PCR-specific primers were designed and synthesised (Table 2). The total RNA extracted from the caecal tissue was reverse transcribed into complementary DNA (cDNA) via an RNA reverse transcription kit (catalogue number: AT311-02). The samples were prepared according to the instructions of the Real-Time PCR Easy™-SYBR Green I Kit (version Number: 1.0-1403). Three negative controls were used for each gene, and *β*-actin was used as the reference gene. The relative quantification of the genes listed in Table 2 was performed via the 2^–ΔΔCT^ method.

**Table 2:** Sequences for real-time PCR primers.

Gene	Accession number	Forward primers (5′-3′)	Reverse primers (5′-3′)	Product size (bp)
*ATAD5*	XM_015279879.4	TGTTCCTTCGTTCCTGGGC	AGATGGCGATTTTCGTGAC	353
ERP29	NM_001276324.1	TACCCTTACGGCGAGAAACA	AAAGTCACCATCCTGGAACAAAT	189
MGST2	XM_001234229.7	GTCTGGTAGTTCTGGCTTGCTTG	CAACTTATGTAATTTCTTCCCAACG	107
HSP90AA1	NM_001109785.2	CAATGGAGGAGGAAGTGGAGAC	GCTTGCTCGGGTCAGTCAAAC	174
PIK3CA	XM_046923916.1	CTCTACAGCTACACCCTACATG	CGACAGCCATTCATTCCATCTG	232
β-Actin	NM_001308613.3	CATCCTGCGTCTGGACCTGG	TAATGTCACGCACGATTTCC	116

### 2.10 Statistical analysis

All experimental data are presented as the mean values. The standard error of the mean (SEM) was determined, and one-way ANOVA was performed via the general linear model (GLM) procedure in SAS software (version 8.02, SAS Institute, Inc., Cary, NC). Dietary berberine treatment and *Salmonella* challenge influenced the gene expression levels and serum parameter data obtained from the 2 × 2 factorial arrangement analysis. Differences at P*p* < 0.05 and *p* < 0.01 were considered statistically significant.

## 3 Results

### 3.1 Effects of berberine and *Salmonella* on the growth performance of chicks

To determine the impact of adding BBR to the diet on the growth performance of *Salmonella*-infected chicks, the average daily gain (ADG), average daily feed intake (ADFI), and feed conversion ratio (FCR) of the chicks in each group at 21 and 35 days of age were calculated based on the obtained data (Table 3). There were no significant differences in the ADG, ADFI, or FCR among the groups from 0 to 35 days of age (*p* > 0.05). These data indicated that throughout the experimental period, neither berberine nor *Salmonella* stimulation significantly affected the average daily weight gain, daily feed intake, or feed conversion ratio of the Sanhuang male chicks.

**Table 3:** Effects of BBR and *Salmonella* on growth performance of chickens^a^ (*N* = 120).

Item^b^	C	B	S	BS	SEM^c^	*p*-value
1–14 days						
ADG (g/bird)	17.64	16.57	15.24	17.33	0.654	0.346
ADFI (g/bird)	23.80	23.45	22.86	22.93	0.226	0.122
FCR (g/g)	1.34	1.42	1.5	1.32	0.062	0.035
15–21 days						
ADG (g/bird)	16.43	17.03	13.51	13.98	0.379	0.437
ADFI (g/bird)	37.46	38.33	37.53	37.65	0.16	0.254
FCR (g/g)	2.31	2.23	2.84	2.84	0.078	0.421
22–35 days						
ADG (g/bird)	32.79	31.15	36.5	36.82	0.161	0.122
ADFI (g/bird)	39.76	38.49	38.67	37.48	0.498	0.441
FCR (g/g)	1.21 ± 0.09	1.22 ± 0.12	1.05 ± 0.12	1.02 ± 0.10	0.042	0.123

a C, Control group; B, Berberine group; S, Positive control group; BS, Berberine-treated group.

b ADG, Average daily gain; ADFI, Average daily feed intake; FCR, Feed conversion rate.

c SEM, Standard error of means.

### 3.2 Effects of BBR and *Salmonella* on the intestinal histomorphology of chicks

**Figure 1:**
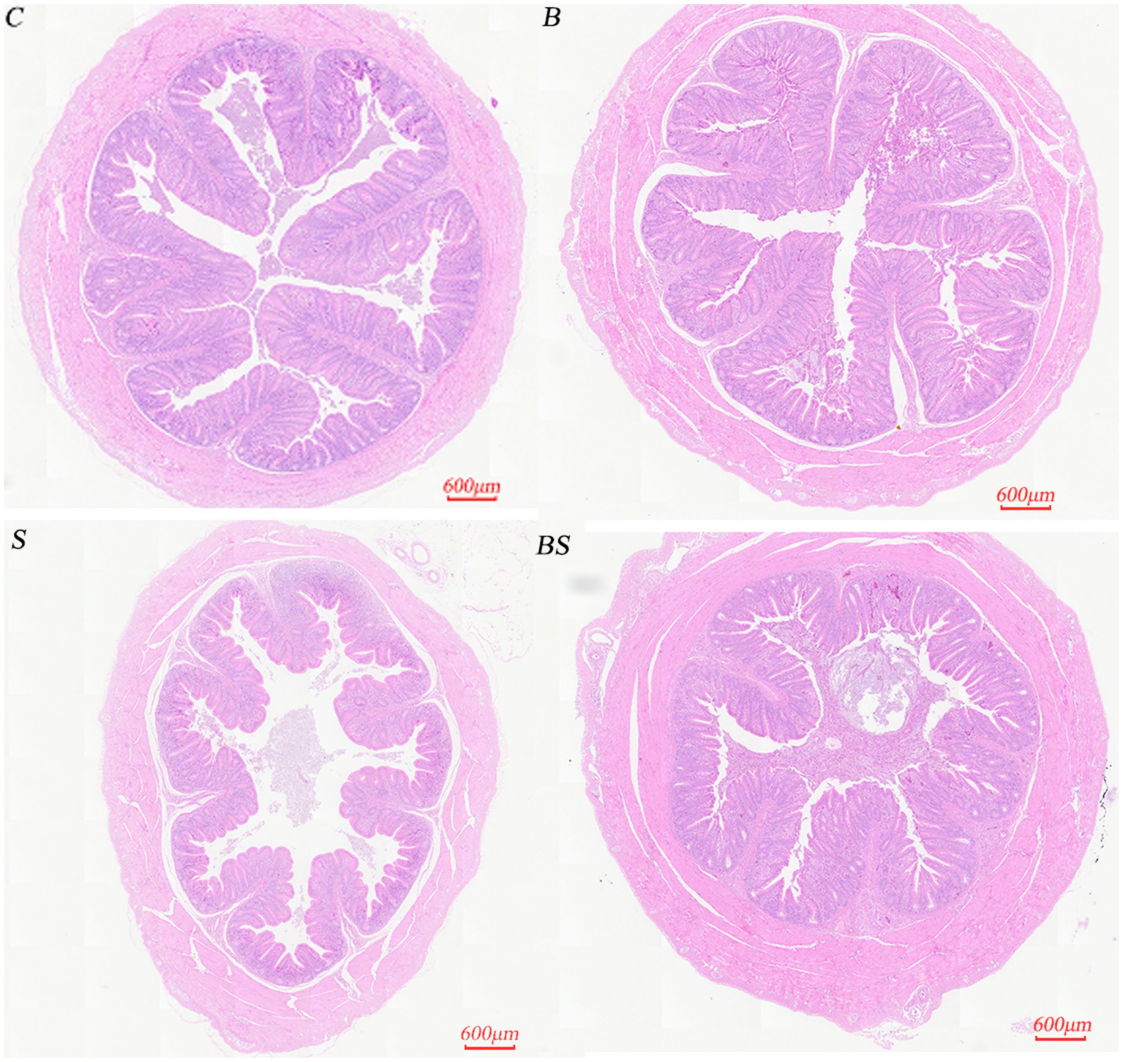
Histological sections of the caecum of a 21-day-old chick (20×). C, Control group; B, Berberine group; S, Positive control group; BS, Berberine-treated group.

To understand the impact of *Salmonella* infection and berberine treatment on the caecal villi and crypts of chicks, as well as to assess the potential role of berberine in mitigating intestinal tissue damage caused by *Salmonella* infection, HE staining was performed on caecal tissues from 21-day-old chicks. Figure 1 shows that there were no significant changes in the morphology of the caecum in the berberine group (B) compared with the control group (C). In contrast, *Salmonella* infection in the positive control group (S) led to a disordered arrangement of caecal epithelial cells and a reduction in villus length, whereas the morphology and villus height in the berberine-treated group (BS) were restored to those of the control group. Table 4 shows variations in the chick caecum’s villus height and crypt depth among the groups. Compared with those in the control group (C), there were changes in the villus height and crypt depth in the BBR group (B). In the *Salmonella*-infected group (S), the villus height decreased by 25.28%, and the crypt depth increased by 27.88%. Compared with the S group, the villus height in the BS group recovered by 29.34% (*p* < 0.05), and the crypt depth in the BS group recovered by 28.17% (*p* < 0.05). These results suggested that berberine alleviates the intestinal tissue damage caused by *Salmonella* infection to a certain extent.

**Table 4:** Effects of BBR and *Salmonella* on Intestinal Histomorphology^1^ (*N* = 12).

Item	C	B	S	BS	SEM^2^	*P*-value
Caecum						
Villus height (μm)	305.7533^a^	294.4500^a^	228.4500^b^	295.4700^a^	12.82137	0.001
Crypt depth (μm)	97.5600^b^	100.9433^b^	124.7600^a^	89.6133^b^	7.38038	0.007

1 C: Control group; B: Berberine group; S: Positive control group; BS: Berberine-treated group.

2 SEM, standard error of means.

a Means with no common superscript within each row are significantly (*p* < 0.05) different.

b Means with no common superscript within each row are significantly (*p* < 0.05) different.

### 3.3 Effects of berberine and *Salmonella* on the content of *Salmonella* in faeces

**Figure 2:**
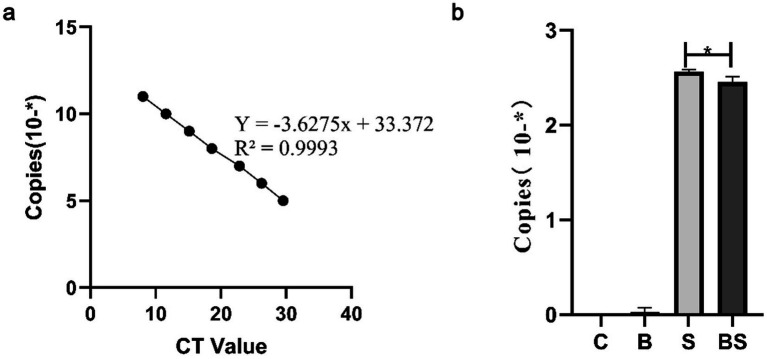
The detection of *Salmonella* content in the faeces of 35-day-old chicks. **(A)** *Salmonella* standard curve; **(B)** Quantitative PCR detection of *Salmonella* content in faeces. *Mean *p* < 0.05.

To determine whether BBR alleviates the presence of *Salmonella* in chicks after intragastric administration of *Salmonella*, faeces were collected from chicks in various groups at 35 days of age for *Salmonella* content determination (Figure 2). Figure 2A shows the *Salmonella* standard curve obtained from the *Salmonella* standard product, and Figure 2B shows the copy number corresponding to the groups of *Salmonella* detected in this test. The content of *Salmonella* in the faeces of chicks significantly increased after intragastric administration of *Salmonella*. However, berberine significantly reduced the content of *Salmonella*.

### 3.4 Effects of berberine and *Salmonella* on immune organ indices in chicks

To evaluate whether berberine alleviates or reverses the negative impact of *Salmonella* infection on indices of immune organs (such as the thymus) in chickens, the liver, spleen, bursa of Fabricius, and thymus from 21-day-old and 35-day-old chicks were weighed. As shown in Table 5, there were slight differences in the liver index, spleen index, and bursa index among the groups, but these differences were not significant (*p* > 0.05). In contrast, there was a substantial change in the thymus index. At 35 days of age, the thymus index in the S group was significantly lower than that in the C group, while the thymus index in the BS group was restored to the level of the control group (*p* < 0.05). These results suggested that adding berberine to the diet mitigates the decrease in the thymus index caused by *Salmonella* infection.

**Table 5:** Effects of BBR and *Salmonella* on immune organ index of chicks^1^ (*N* = 20).

Item	C	B	S	BS		*p*	
					Berberine	*Salmonella*	Interaction
0–21 days							
Liver index (g/kg)	31.46	31.04	33.33	32.62	0.041	0.041	0.223
Thymus index (g/kg)	4.08^b^	3.34 ^b^	3.46^b^	2.34^a^	0.539	0.222	<0.001
Spleen index (g/kg)	1.57	1.58	1.58	1.50	0.434	0.233	0.532
Bursa index (g/kg)	3.04	3.25	2.83	2.87	0.651	0.788	0.760
22–35 days							
Liver index (g/kg)	32.39	29.07	33.29	32.40	0.176	0.203	0.233
Thymus index (g/kg)	3.99^b^	3.05^b^	2.57^a^	3.83^b^	0.242	<0.001	0.539
Spleen index (g/kg)	2.07	1.81	2.18	1.87	0.124	0.233	
Bursa index	2.84	2.88	2.84	3.03	0.419	0.735	0.687

1 C, Control group; B, Berberine group; S, Positive control group; BS, Berberine-treated group.

a Means with no common superscript within each row are significantly (*p* < 0.05) different.

b Means with no common superscript within each row are significantly (*p* < 0.05) different.

### 3.5 Effects of berberine and *Salmonella* on biochemical indices of chicks

**Figure 3:**
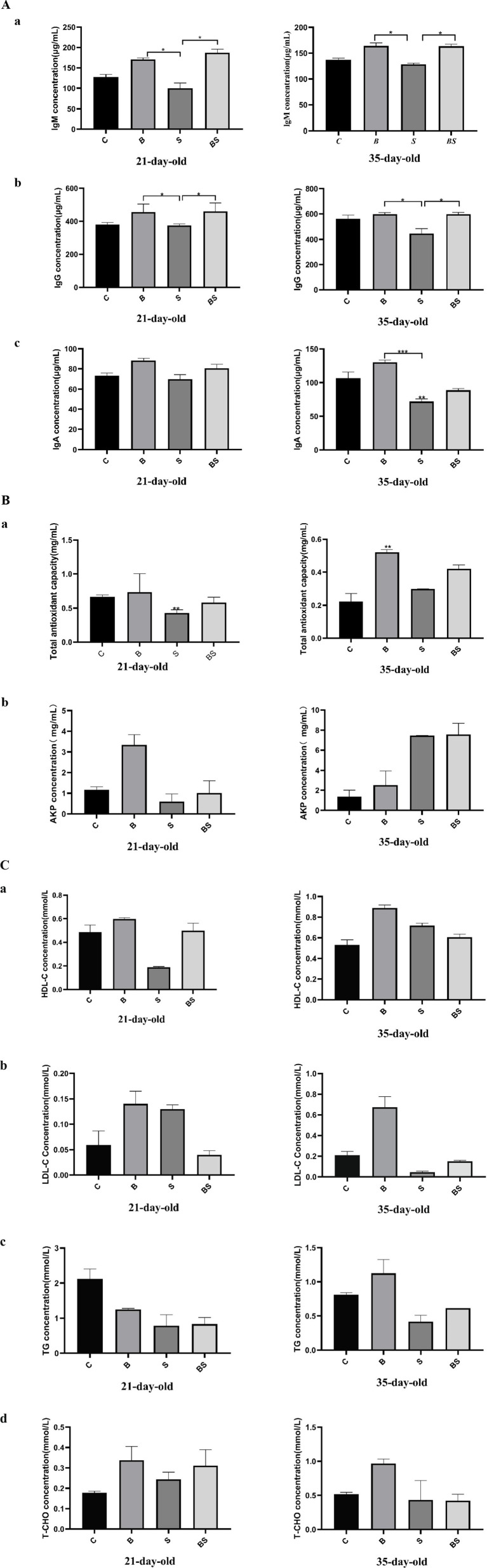
Effects of BBR and *Salmonella* on serum biochemical indexes of chickens. C, Control group; B, Berberine group; S, Positive control group; BS, Berberine-treated group. **(*a*)** Effects of BBR and *Salmonella* on IgM; **(*b*)** Effects of BBR and *Salmonella* on IgG; **(*c*)** Effects of BBR and *Salmonella* on IgA. *Mean *p* < 0.05; **Mean *p* < 0.01; ***Mean *p* < 0.001. **(*a*)** Effects of BBR and *Salmonella* on total antioxidant capacity; **(*b*)** Effects of BBR and *Salmonella* on alkaline phosphatase. *Mean *p* < 0.05; **Mean *p* < 0.01; ***Mean *p* < 0.001. **(*a*)** Effects of BBR and *Salmonella* on high-density lipoprotein; **(*b*)** Effects of BBR and *Salmonella* on low-density lipoprotein; **(*c*)** Effects of BBR and *Salmonella* on triglycerides; **(*d*)** Effects of BBR and *Salmonella* on total cholesterol. *Mean *p* < 0.05; **Mean *p* < 0.01; ***Mean *p* < 0.001.

To explore whether berberine modulates the body’s response to *Salmonella* infection, the levels of immunoglobulins (IgA, IgM, and IgG), antioxidant indicators (alkaline phosphatase activity and total antioxidant capacity), high-density lipoprotein (HDL), low-density lipoprotein (LDL), triglycerides, and total cholesterol were measured in the serum of chicks from each group at 21 and 35 days of age. Compared with those in the control group (C), there was an increasing trend in the concentrations of IgA, IgM, and IgG in the B group (Figure 3A). Still, IgA, IgM, and IgG concentrations were lower in the *Salmonella*-infected group (S). Compared with those in the S group, IgA, IgM, and IgG concentrations in the BS group were more remarkable, with significant increases in the IgM and IgG concentrations (*p* < 0.05). The effects of berberine and *Salmonella* on serum antioxidant indicators are shown in Figure 3B. At 21 days of age, compared with that in the C group, the total antioxidant capacity in the S group significantly decreased (*p* < 0.05), and the concentration of alkaline phosphatase also reduced, which was mitigated in the BS group. At 35 days of age, compared with that in the C group, the total antioxidant capacity in the B group significantly increased (*p* < 0.05). Compared with that in the B group, the total antioxidant capacity in the S group decreased, which was mitigated in the BS group. Compared with those in the C group, the concentrations of high-density lipoprotein (HDL) and low-density lipoprotein (LDL), as well as the total cholesterol content, were elevated in the B group; however, the opposite was observed after *Salmonella* gavage, but no significant differences were observed (*p* > 0.05) (Figure 3C). These results indicated that BBR modulates various biochemical indicators in the body after *Salmonella* infection.

### 3.6 Effects of berberine and *Salmonella* on the intestinal microbial flora of chicks

To explore the protective effects of adding berberine to the diet on *Salmonella*-induced enteritis and gut microbiota dysbiosis in chicks, caecal contents from 21-day-old chicks were sent to Shanghai Majorbio Bio-Pharm Technology Co., Ltd. for 16S rRNA sequencing of the gut microbiota. After rarefying the gut microbiota sequences to 29,026 sequence counts for each group, *α*-diversity analysis was conducted, including species diversity indices (Shannon index and Simpson index) and species richness indices (Sobs, Ace, and Chao indices) (Table 6). The Sobs and Chao indices in both the S group and BS group were significantly lower than those in the C group (*p* < 0.05), indicating that *Salmonella* gavage reduced the variety and quantity of the gut microbial populations in the chicks.

**Table 6:** Intestinal flora of broilers α-diversity (*N* = 12).

	Processing group^1^			
Item	C	B	S	BS
Sobs	401.33 ± 12.22^b^	397.67 ± 18.58^a^^b^	367.67 ± 24.21^a^	370 ± 7.81^a^
Shannon	3.84 ± 0.54	3.93 ± 0.45	3.96 ± 0.23	4.12 ± 0.20
Simpson	0.08 ± 0.08	0.07 ± 0.04	0.06 ± 0.03	0.03 ± 0.01
Ace	432.38 ± 18.79^b^	430.91 ± 21.45^a^^b^	396.6 ± 29.98^a^^b^	395.76 ± 10.34^a^
Chao	435.83 ± 23.05^b^	436.34 ± 22.56^b^	394.52 ± 29.87^a^	394.36 ± 7.30^a^
Coverage	0.99 ± 0.01	0.99 ± 0.01	0.99 ± 0.01	0.99 ± 0.01

1 C, Control group; B, Berberine group; S, Positive control group; BS, Berberine-treated group.

a Means with no common superscript within each row are significantly (*p* < 0.05) different.

b Means with no common superscript within each row are significantly (*p* < 0.05) different.

**Figure 4:**
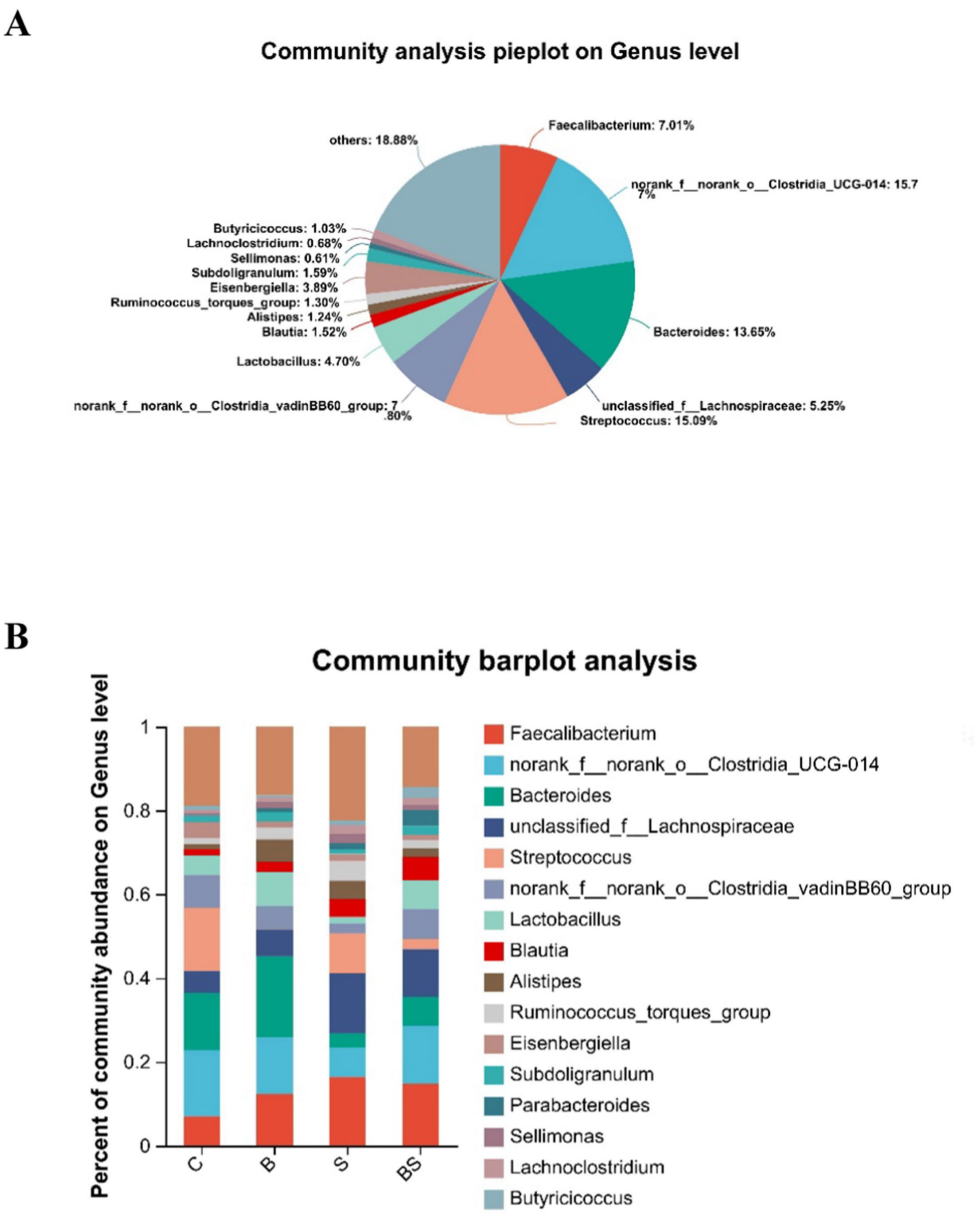
Gut microbiota composition. **(A)** Community composition at the genus level. Different colours represent distinct bacterial genera, and the area of the pie chart segments corresponds to the percentage that each genus constitutes within the community. **(B)** Variation in dominant genera composition across different groups. The bars depicted in various colours represent different bacterial genera, with the length of the bars indicating the proportional representation of each genus.

Analysis of the composition of the caecal microbiota across the groups revealed 16 predominant bacterial genera (Figure 4A), including Faecalibacterium, norank_f__norank_o__Clostridia_UCG-014, Bacteroides, unclassified_f__Lachnospiraceae, Streptococcus, norank_f__norank_o__Clostridia_vadinBB60_group, Lactobacillus, Blautia, Alistipes, Ruminococcus_torques_group, Eisenbergiella, Subdoligranulum, Parabacteroides, Sellimonas, Lachnoclostridium, and Butyricicoccus.

Compared with those in the control group (C), adding BBR increased the abundance of several genera, including Faecalibacterium, Bacteroides, Lactobacillus, and Alistipes. Still, it reduced the abundance of Streptococcus and Eisenbergiella (Figure 4B). Compared with those in the control group (C), the abundances of Faecalibacterium, unclassified_f__Lachnospiraceae, Blautia, and Alistipes were more significant in the *Salmonella*-infected group (S). In contrast, the abundances of norank_f__norank_o__Clostridia_UCG-014, Bacteroides, Streptococcus, norank_f__norank_o__Clostridia_vadinBB60_group, and Lactobacillus were lower in the S group. Compared with those in the S group, the abundances of norank_f__norank_o__Clostridia_UCG-014, Bacteroides, norank_f__norank_o__Clostridia_vadinBB60_group, and Lactobacillus were more significant in the BS group. On the contrary, the abundances of Faecalibacterium, Streptococcus, and Alistipes were lower in the BS group. These results indicated that *Salmonella* gavage increases the levels of harmful bacterial genera in the gut. In contrast, adding berberine to the feed reduces the levels of harmful bacterial genera and increases the levels of beneficial bacterial genera.

### 3.7 Transcriptome sequencing and bioinformatics analysis

**Figure 5:**
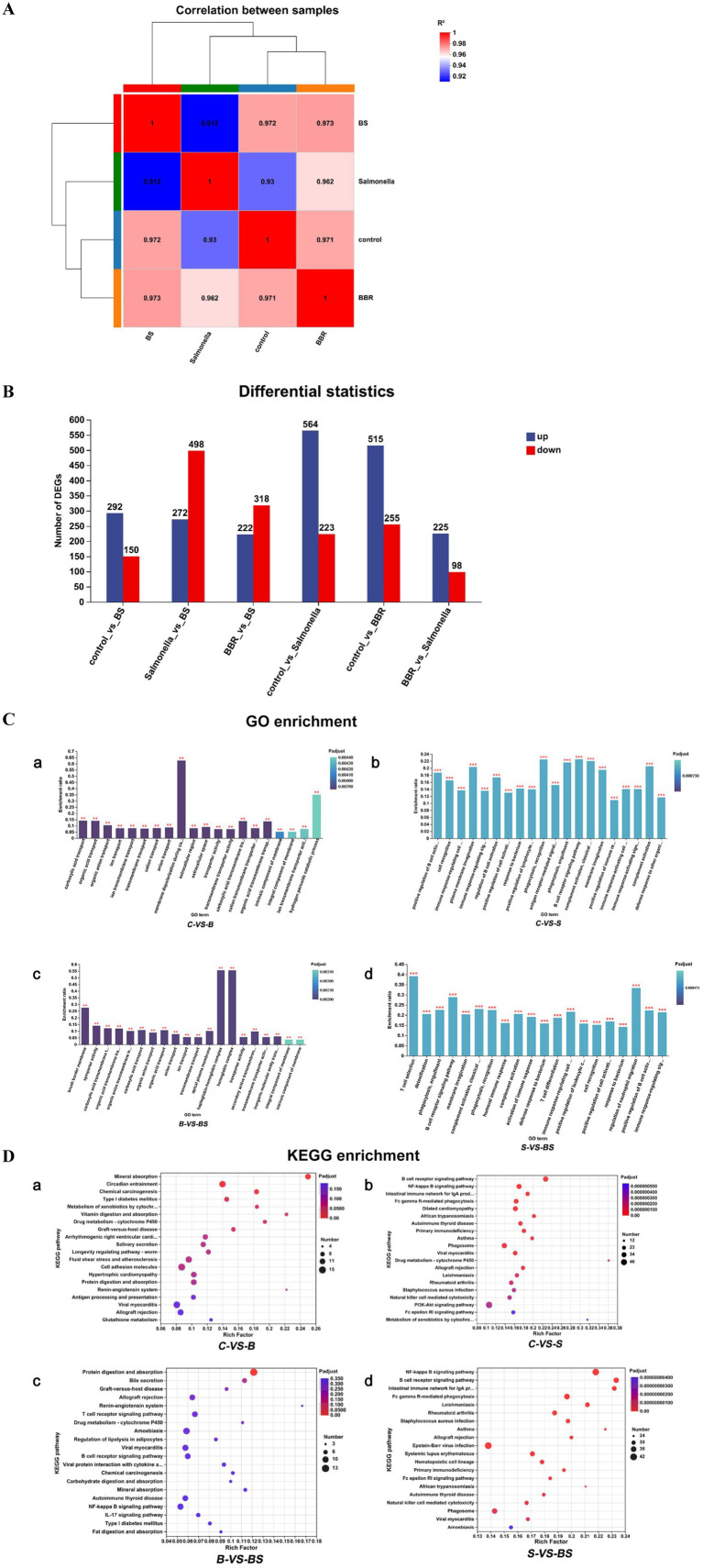
Transcriptome sequencing result analysis. **(A)** Sample correlation analysis. The right and bottom sides of the chart list the sample names, while the left and upper sides depict the clustering of the samples. The coloured squares represent the degree of correlation between two samples, with different colours indicating a higher or lower correlation. **(B)** Statistical chart of expression difference between groups. **(C)** GO Enrichment Analysis. The horizontal axis represents the GO term, and the vertical axis represents the enrichment factor. The gradient of the bar colour indicates the significance of the enrichment, *Mean *p* < 0.05; **Mean *p* < 0.01; ***Mean *p* < 0.001. **(D)** KEGG enrichment analysis. The vertical axis represents the pathway name, and the horizontal axis represents the enrichment factor. The size of the dots indicates the number of genes in a particular pathway, while the colour of the dots corresponds to different *p*_adjust_ ranges.

To further explore the molecular mechanisms of BBR treatment for *Salmonella*-induced enteritis in chicks, the caecum from 21-day-old chicks was collected and sent to Shanghai Majorbio Bio-Pharm Technology Co., Ltd. for transcriptome sequencing. A total of 96.93 Gb of clean data were obtained from the sequencing, with each sample yielding more than 6.1 Gb of clean data. The percentage of Q30 bases was more significant than 93.82%, and a total of 20,577 genes were detected, including 19,472 known genes. Figure 5A shows that the correlation coefficients among all samples are high, ranging from 0.912 to 0.973, indicating a strong correlation between these samples. The differential gene expression analysis among the groups is shown in Figure 5B. Compared with the control group (C), the BBR group (B) had 515 upregulated genes and 255 downregulated genes. Compared with the control group, the *Salmonella*-infected group (S) had 564 upregulated genes and 223 downregulated genes. Compared with the *Salmonella*-challenged group (S), the BBR-treated group (BS) had 272 upregulated genes and 498 downregulated genes.

According to the GO enrichment results (Figure 5C), compared with those in the control group (C), the differentially expressed genes (DEGs) in the *Salmonella* group (S) were enriched predominantly in the following biological processes: positive regulation of B cell activation, plasma membrane invagination, phagocytosis recognition, phagocytosis engulfment, the B cell receptor signalling pathway, complement activation, and the classical pathway (Figure 5C-b). Compared with those in the *Salmonella* group (S), the DEGs in the berberine-treated group (BS) were enriched mainly in T cell selection, the B cell receptor signalling pathway, regulation of neutrophil migration, and positive regulation of B cell activation (Figure 5C-d).

The KEGG pathway enrichment analysis results (Figure 5D) revealed that, compared with those in the control group (C), the DEGs in the berberine group (B) were enriched primarily in the following pathways: mineral absorption, chemical carcinogenesis, vitamin digestion and absorption, drug metabolism (cytochrome P450), and graft vs. host disease (Figure 5D-a). Compared with those in the control group (C), the DEGs in the *Salmonella* group (S) were enriched mainly in pathways related to the B cell receptor signalling pathway, the NF-*κ* B signalling pathway, the intestinal immune network for IgA production, African trypanosomiasis, drug metabolism (cytochrome P450), and asthma (Figure 5D-b). Compared with those in the berberine group (B), the differentially expressed genes in the berberine-treated group (BS) were predominantly enriched in the following pathways: protein digestion and absorption; renin–angiotensin system; and mineral absorption (Figure 5D-c). Compared with those in the *Salmonella* group (S), the DEGs in the berberine-treated group (BS) were enriched mainly in the following pathways: the NF-κ B signalling pathway, the B cell receptor signalling pathway, the intestinal immune network for IgA production, asthma, and the African trypanosomiasis pathway (Figure 5D-d).

### 3.8 Quantitative real-time PCR analysis

**Figure 6:**
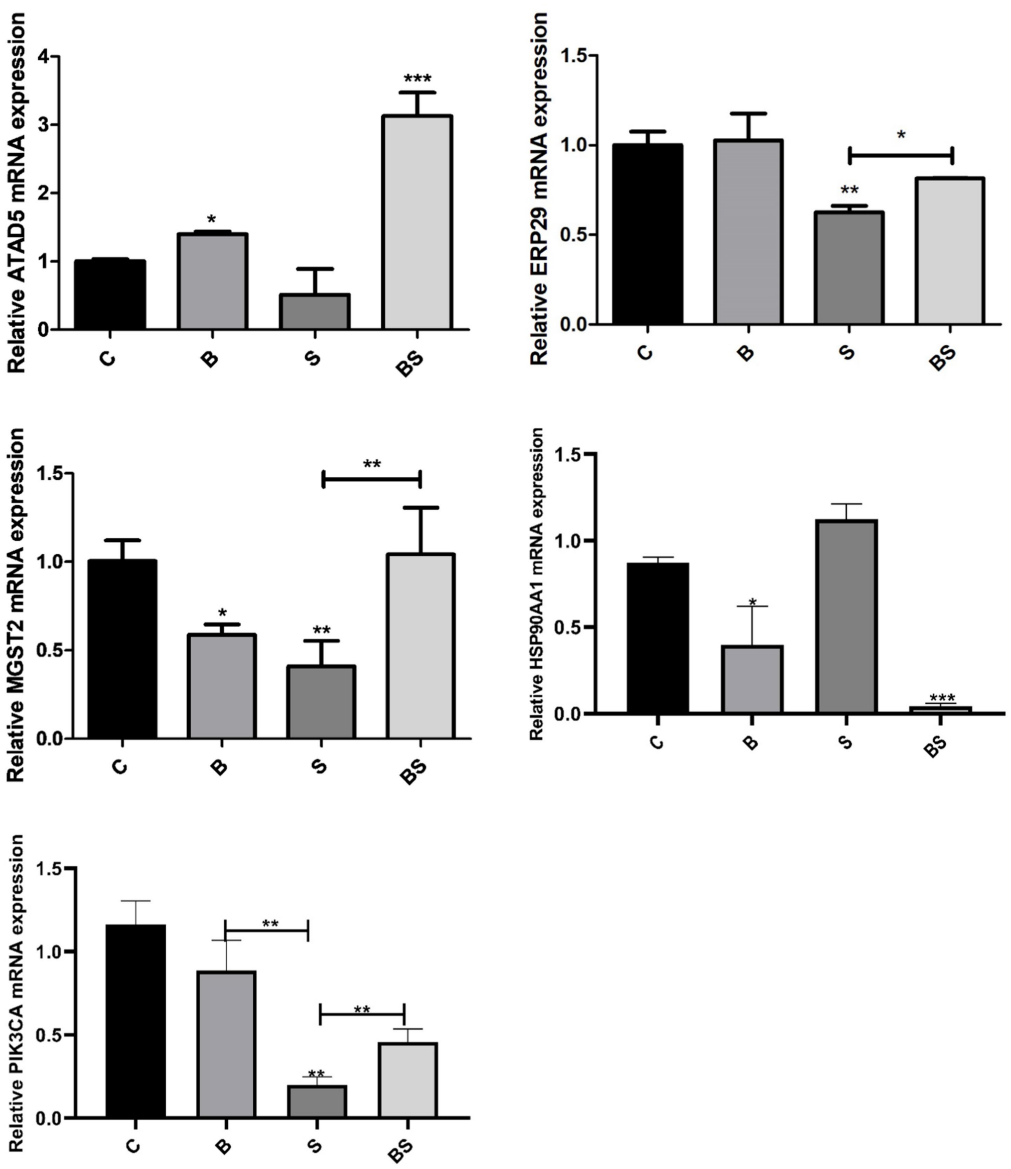
Effects of Berberine on the expression of ATAD5, ERP29, MGST2, HSP90AA1, and PIK3CA genes in the caecum of broilers. *Mean *p* < 0.05; **Mean *p* < 0.01; ***Mean *p* < 0.001.

The transcriptome sequencing results were verified via quantitative real-time PCR analysis and gene expression detection. On the basis of the results of the transcriptome sequencing, five genes whose expression significantly differed were selected, namely, ATAD5, ERP29, MGST2, HSP90AA1, and PIK3CA, and verified using quantitative real-time PCR (Figure 6). Compared with those in the control group, the expression levels of ATAD5, ERP29, MGST2, and PIK3CA were downregulated in the *Salmonella*-challenged group (S) and upregulated in the berberine-treated group (BS), whereas the opposite trend was observed for the expression of HSP90AA1.

## 4 Discussion

In recent years, berberine has been included in the Chinese Pharmacopoeia because of its anti-inflammatory, antioxidant, antibacterial, and antiapoptotic properties, and it has garnered widespread attention in the prevention and treatment of gastrointestinal diseases (Song et al., 2020). The present study revealed that the addition of 120 mg/kg berberine to the diet had no significant effect on the average daily gain (ADG), average daily feed intake (ADFI), or feed conversion ratio (FCR) of the broiler chicks aged 0–35 days (*p* > 0.05), which may be due to the different growth stages of the broilers or that the feeding period was too short. In addition, research has confirmed that the appropriate addition of berberine to the diet improves the growth performance of poultry. Zhu et al. reported that adding 250 mg/kg berberine to the diet effectively enhances the growth performance of broilers at 63 days of age. Zhang et al. reported that adding 100 mg/kg berberine to the diet effectively improves the growth performance of broilers at 42 days of age (Zhang et al., 2013; Zhu et al., 2021).

Villus height and crypt depth are essential indicators for assessing the intestinal tissue health because they directly reflect intestinal epithelial cells’ renewal and regenerative capacity. Villi are the main structures involved in nutrient absorption in the intestine, whereas crypts are the locations of intestinal stem cells and are responsible for generating new epithelial cells. When the intestine is infected or damaged, these structures may change, such as a reduction in villus height and an increase in crypt depth. These changes are usually associated with a decline in intestinal function and a disease state (Jing et al., 2021). In the present study, the villus height in the S group (*Salmonella*-infected) was significantly reduced, and the S group’s crypt depth was significantly increased. The villus height and crypt depth in the BS group (Berberine-treated) were both restored compared with those in the S group. This may be due to BBR directly inhibiting or killing invading *Salmonella* in the intestine or to BBR repairing epithelial cells, thereby alleviating the intestinal structural damage caused by *Salmonella*. Berberine has antibacterial properties and can directly inhibit or kill bacteria, reducing pathogen attacks on the intestine (Sun et al., 2023). In addition, BBR promotes the repair and regeneration of intestinal epithelial cells, helping to restore the typical structure and function of the intestine (Huo and Wang, 2024).

Immune organs govern the proliferation and development of immune cells. The weights of the thymus, spleen, and bursa of Fabricius, representing the most critical immune organs in poultry, increased, which reflected the growth and reproduction of immune cells, further indicating an increase in the immune capabilities of the organism. The present study revealed that after broilers were inoculated with 1.2 × 10^9^ CFU/mL *Salmonella*, there was a significant decrease in the thymus index of 35-day-old chicks, suggesting that the invasion of *Salmonella* decreased the immune system performance in broilers. Wang Shasha et al. also confirmed that the thymus index of chicks significantly decreases after inoculation with 1 × 10^9^ CFU/mL *Salmonella* for 21 days (Xian-Yao, 2015). Immunoglobulins are an essential part of the immune system in animals, and IgM, IgA, and IgG are essential indicators for measuring humoural immune responses. Higher levels of these immunoglobulins indicate stronger immune functions in the organism (Jili, 2011). The present study revealed that at 21 days of age, the concentrations of IgM and IgG in the serum of chicks in the treatment group were significantly greater than those in the serum of chicks in the challenge group, indicating that the addition of BBR to feed reversed the decrease in immune function caused by *Salmonella* infection in chicks. Oxidative stress is a negative impact caused by the production of free radicals, such as hydroxyl radicals and superoxide anions, within the animal body. Excessive reactive oxygen species damage proteins and nucleic acids, as well as produce large amounts of MDA to injure tissues, and they cause inflammation and disease in organisms (He Yun and Xiaoying, 2020). At 35 days of age, the addition of berberine significantly increased the total antioxidant capacity in the serum of chicks, which was consistent with previously published results (Zhu Cui et al., 2022).

16S ribosomal RNA (16S rRNA) is a crucial component of the ribosome’s small subunit (30S subunit) in prokaryotes, including bacteria and archaea. 16 rRNA plays a significant role in bacterial taxonomy, phylogenetic analysis, and microbial community research. The sequence of the 16S rRNA gene contains both highly conserved regions and highly variable regions, making it an ideal molecular marker for studying microbial diversity and evolutionary relationships (Nelson et al., 2014). In the present study, caecal contents from 21-day-old chicks were sent to a Shanghai Majorbio Bio-Pharm Technology Co., Ltd. for 16S rRNA sequencing of the gut microbiota. *α*-Diversity assessment reflects the microbial community’s richness and diversity, including a series of statistical analysis indices to estimate species abundance and diversity within environmental communities. The Sobs, Ace, and Chao indices of the *Salmonella*-challenged and the berberine-treated groups were significantly lower than those of the control group, indicating that *Salmonella* gavage effectively reduced the microbial abundance of the caecal microbiota. Similarly, Wang et al. revealed that *Salmonella* reduces the microbial abundance of the gut microbiota (Chen and Stappenbeck, 2019).

The genus-level gut microbiota diversity results revealed that the addition of berberine to the diet increased the abundance of genera, such as Faecalibacterium, Bacteroides, and Lactobacillus, but reduced the abundance of other genera, such as Streptococcus and Eisenbergiella. In contrast, gavage with *Salmonella* led to an increase in the abundance of some genera, such as Faecalibacterium, Blautia, and Alistipes, but a decrease in the abundance of Bacteroides, Streptococcus, and Lactobacillus. The genus Lactobacillus plays an important role in maintaining the health of the animal body. Lactobacillus enhances the absorption of nutrients by the body, whereas it inhibits the proliferation of pathogenic bacteria in the intestine. The gut is protected from harmful bacteria by the competitive exclusion of pathogens and the production of antimicrobial substances, which are probiotics of the gut (Rajab et al., 2020). The genus Faecalibacterium promotes the production of short-chain fatty acids (such as butyrate), which are crucial for maintaining the health of intestinal epithelial cells and strengthening intestinal barrier function (Jiang et al., 2024). The genus Streptococcus is widely distributed in nature, and the majority of the species of Streptococcus are nonpathogenic; however, Group A Streptococcus has strong pathogenicity, and Gallid Streptococcus causes causing streptococcosis in chickens, which can severely damage the economic benefits of breeders (Barnham et al., 1980; Wollein Waldetoft and Råberg, 2014). Compared with those in the *Salmonella*-challenged group (S), the abundances of Bacteroides and Lactobacillus in the berberine-treated group (BS) increased, whereas the abundances of Faecalibacterium, Streptococcus, and Alistipes decreased in the BS group. These results indicated that BBR alleviates the negative effects of *Salmonella* infection by regulating the balance of the gut microbiota, which agreed with previously published findings (Zhang et al., 2013).

Transcriptome sequencing is a rapid, efficient, and accurate method for identifying differences in gene expression levels among various samples. The present study sent caecal tissues from 21-day-old chicks to Shanghai Majorbio Bio-Pharm Technology Co., for transcriptome sequencing. This study aimed to screen for key genes that may be involved in the alleviation of *Salmonella* infection in chicks by berberine and to explore the molecular mechanisms by which berberine alleviates *Salmonella* infection in chicks at the mRNA level. Compared with those in the blank control group (C), there were 787 DEGs in the *Salmonella*-challenged group (S). These genes were significantly enriched in the following GO terms: positive regulation of B cell activation, plasma membrane invagination, phagocytosis recognition, phagocytosis engulfment, the B cell receptor signalling pathway, and complement activation of the classical pathway. The enrichment of ‘positive regulation of B-cell activation’ and the ‘B cell receptor signalling pathway’ was related to immune system processes, indicating that *Salmonella* infection may activate B cell-mediated immune responses, in which B cells play a crucial role in antibody production and the formation of immune memory (Okkenhaug, 2016; Wang and Zheng, 2013). The enrichment of ‘phagocytosis recognition’ and ‘phagocytosis engulfment’ is related to phagocytosis, suggesting that *Salmonella* infection may increase the phagocytic activity of phagocytes (such as macrophages and neutrophils), which is an essential mechanism by which the host clears pathogens (Jiang et al., 2021; Wang and Ding, 2020). The enrichment of the ‘complement activation’ and ‘classical pathway’ indicated that the complement system is activated after *Salmonella* infection, which is part of the host’s innate immunity and plays a vital role in pathogen clearance and inflammatory responses (Bajic et al., 2015; Noris and Remuzzi, 2013). Compared with those in the *Salmonella*-challenged group (S), there were 770 DEGs in the berberine-treated group (BS), which were enriched mainly in GO terms related to T cell selection, the B cell receptor signalling pathway, the regulation of neutrophil migration, and the positive regulation of B cell activation. The enrichment of ‘T cell selection’ suggested that berberine may affect the T cells’ selection and maturation process, which is crucial for the development of adaptive immune responses (Turner et al., 2009). The enrichment of ‘regulation of neutrophil migration’ suggested that berberine may have a regulatory effect on the migration of neutrophils, which is essential for the localization and control of inflammatory responses (Brazil and Parkos, 2016). Moreover, the enrichment of the ‘B cell receptor signalling pathway’ and ‘positive regulation of B cell activation’ indicated that berberine might alleviate the negative effects of *Salmonella* infection in chicks by modulating the function of B cells and antibody production.

KEGG enrichment analysis was performed on the significantly differentially expressed genes. Compared with those in the control group (C), the DEGs in the *Salmonella*-infected group (S) were enriched predominantly in the following pathways: the B cell receptor signalling pathway, NF-*κ* B signalling pathway, intestinal immune network for IgA production, African trypanosomiasis, drug metabolism (cytochrome P450), and asthma pathways. The enrichment of the ‘B-cell receptor signalling pathway’ and ‘intestinal immune network for IgA production’ pathways highlighted the impact of *Salmonella* infection on the intestinal immune network, which may be related to the host’s defence mechanisms against pathogens. IgA is the most abundant type of antibody in the mucosal immune response of the gut. The enrichment of this pathway may reflect the impact of *Salmonella* infection on IgA production in the intestinal mucosa, which is crucial for local immune defence and pathogen clearance (Gutzeit et al., 2014). The enrichment of the ‘NF–κ B signalling pathway’ suggested that infection may activate the NF-κB pathway, which is a key transcription factor that regulates the expression of inflammatory genes (Hayden and Ghosh, 2012). *Salmonella* infection may induce the expression of inflammatory genes and trigger the host inflammatory response by activating the NF-κB pathway. Compared with those in the S group, the DEGs in the berberine-treated group (BS) were enriched mainly in the following pathways: the NF–κ B signalling pathway, B cell receptor signalling pathway, the intestinal immune network for IgA production, asthma pathway, and African trypanosomiasis pathway. The enrichment of the ‘NF–κ B signalling pathway’, ‘B cell receptor signalling pathway’, and ‘intestinal immune network for IgA production’ suggested that the addition of berberine to the diet may regulate the upregulation or downregulation of key genes in these pathways, thereby balancing the inflammatory and immune responses induced by *Salmonella* infection. The results of the GO and KEGG enrichment analyses suggested that berberine may alleviate the negative effects of *Salmonella* infection in chicks by regulating immune responses, enhancing phagocytosis, affecting complement activation, modulating drug metabolism, and modulating digestive absorption mechanisms.

Based on the transcriptome data, five genes with significantly differential expression, namely, ATAD5, ERP29, MGST2, PIK3CA, and HSP90AA1, were selected and qRT–PCR was used to measure their mRNA expression levels in the caecal tissues of broilers after *Salmonella* challenge. ATAD5 is a member of the nucleotide adenosine triphosphatase (ATPase) family involved in DNA replication and cell cycle control. ATAD5 interacts with proliferating cell nuclear antigen (PCNA) to facilitate normal DNA replication and cell division. Studies have shown that downregulation of ATAD5 may be associated with DNA replication stress and genomic instability (Kim et al., 2020). According to the present results, the downregulation of ATAD5 after *Salmonella* infection may indicate an impaired cell cycle and DNA replication in the intestine, and berberine may alleviate this obstruction. Endoplasmic reticulum protein 29 (ERp29) is a molecular chaperone that plays a crucial role in protein folding, transport, and secretion (Ye et al., 2017). In the present study, the ERP29 gene expression level was downregulated after *Salmonella* infection in chicks, and this downregulation was partially reversed by berberine treatment. Thus, berberine may block the association between *Salmonella* invasion and ERP29, protecting the tight junctions of intestinal tissue, which is also supported by the present histopathological results and previous laboratory findings (McLaughlin et al., 2018). Phosphatidylinositol-4,5-bisphosphate 3-kinase, catalytic subunit *α* (PIK3CA) is a crucial component of the phosphatidylinositol-3-kinase (PI3K) signalling pathway. Studies have shown that this pathway plays a vital role in various biological processes within the cell, including cell survival, proliferation, apoptosis, metabolism, and cell migration (Iorga et al., 2017). The overexpression or activation of PIK3CA often leads to abnormal activation of the PI3K–Akt pathway, which may be related to cell survival, apoptosis, and oxidative stress status (Engelman et al., 2006). In the present study, berberine regulated the gene expression of PIK3CA, suggesting that berberine may have an inhibitory effect on the PIK3CA/PI3K-Akt signalling pathway by reducing its abnormal activation, which may help regulate cell proliferation and survival, as well as reduce the risk of apoptosis (Manning, 2017; Vanhaesebroeck et al., 2010). HSP90AA1 is closely related to inflammation and regulates cell proliferation and apoptosis. Previous studies have shown that inhibiting HSP90AA1 induces autophagy, thereby improving ulcerative colitis (Song et al., 2020; Yang et al., 2020). Su et al. revealed that berberine has functions similar to those of HSP90 inhibitors and inhibits the expression of the HSP90 protein in colon cancer SW840 cells, thereby inhibiting the proliferation of SW840 cells (Su et al., 2015).

Based on the above findings, the prevention and treatment of *Salmonella* infection by berberine may be related to the coordinated action of multiple targets. Berberine is likely involved in treating *Salmonella* infection by regulating cellular processes, such as the cell cycle, apoptosis, oxidative stress, and inflammatory responses.

## 5 Conclusion

The present study revealed that berberine has a promising therapeutic effect on *Salmonella* infection in chicks. Moreover, berberine regulates the expression of multiple genes involved in several pathways. These results suggest that BBR treatment of *Salmonella* has potential clinical value, and its specific molecular mechanisms warrant further in-depth investigation.

## Data Availability

The datasets presented in this study can be found in online repositories. The names of the repository/repositories and accession number(s) can be found in the article/supplementary material.
